# Do calcium channel blockers applied to cardiomyocytes cause increased channel expression resulting in reduced efficacy?

**DOI:** 10.1038/s41540-024-00347-3

**Published:** 2024-03-01

**Authors:** Karoline Horgmo Jæger, Verena Charwat, Samuel Wall, Kevin E. Healy, Aslak Tveito

**Affiliations:** 1 Simula Research Laboratory, Oslo, Norway; 2 Organos Inc., Berkeley, CA USA; 3 Department of Bioengineering, University of California, Berkeley, CA USA; 4 Department of Material Science and Engineering, University of California, Berkeley, CA USA

**Keywords:** Cell biology, Biomarkers, Computer modelling, Nonlinear dynamics, Computational biology and bioinformatics

## Abstract

In the initial hours following the application of the calcium channel blocker (CCB) nifedipine to microtissues consisting of human induced pluripotent stem cell-derived cardiomyocytes (hiPSC-CMs), we observe notable variations in the drug’s efficacy. Here, we investigate the possibility that these temporal changes in CCB effects are associated with adaptations in the expression of calcium ion channels in cardiomyocyte membranes. To explore this, we employ a recently developed mathematical model that delineates the regulation of calcium ion channel expression by intracellular calcium concentrations. According to the model, a decline in intracellular calcium levels below a certain target level triggers an upregulation of calcium ion channels. Such an upregulation, if instigated by a CCB, would then counteract the drug’s inhibitory effect on calcium currents. We assess this hypothesis using time-dependent measurements of hiPSC-CMs dynamics and by refining an existing mathematical model of myocyte action potentials incorporating the dynamic nature of the number of calcium ion channels. The revised model forecasts that the CCB-induced reduction in intracellular calcium concentrations leads to a subsequent increase in calcium ion channel expression, thereby attenuating the drug’s overall efficacy. The data and fit models suggest that dynamic changes in cardiac cells in the presence of CCBs may be explainable by induced changes in protein expression, and that this may lead to challenges in understanding calcium based drug effects on the heart unless timings of applications are carefully considered.

## Introduction

Excitable cells exhibit electrochemical homeostasis over extended periods, even though the constituent membrane proteins (e.g., ion channels), which underpin the action potential of these cells, are in a continuous state of renewal. How is the expression of ion channels controlled to preserve the electrical properties essential for physiological functions? This pressing question has been the subject of extensive investigation by Marder and colleagues, among others, over several years; see, e.g., refs. 1–7.

In one significant contribution, O’Leary et al.^4^ developed a mathematical framework to represent ion channel expression. Their work was founded on the hypothesis that the intracellular calcium concentration governs ion channel expression levels. According to their model, if the calcium concentration falls below a specific target value, the number of ion channels will, through a multistep process of DNA transcription, RNA translation, and protein trafficking, increase until that target value is met.

Their modeling framework has found applications beyond the original scope. Recently, Moise and Weinberg^8^ adapted this model to analyze ion conductances within the cells of the Sinoatrial node, further demonstrating the model’s relevance and utility in understanding the complex dynamics of ion channel regulation; see also ref. ^9^.

One version of the model for dynamic ion channel expression can be written in the following form,

1 $${\tau }_{m}\frac{dm}{dt}={c}^{* }-c,$$

2 $${\tau }_{n}\frac{dn}{dt}=m-n.$$

Here, *m* and *n* are relative changes in the number of messenger RNAs (mRNAs) and the number of expressed ion channels proteins, respectively (*m* = *n* = 1 is the default case). Furthermore, *τ*_*m*_ and *τ*_*n*_ are time constants, *c* is the cytosolic calcium concentration, and c* is the target level of the cytosolic calcium concentration. In this model, we let *M*_0_ and *N*_0_ represent the default numbers of mRNAs and ion channel proteins, respectively. The associated dynamic numbers are given by *M*(*t*) = *m*(*t*)*M*_0_ and *N*(*t*) = *n*(*t*)*N*_0_, where the evolution of the relative changes *m* and *n* are governed by the system (1) and (2). In the computations of this study, we only let the number of L-type calcium channels be regulated by the model (1) and (2) and let the number of the remaining types of channels be fixed. The associated calcium current is given by

3 $${I}_{{{{\rm{CaL}}}}}=\frac{n{N}_{0}}{A{C}_{m}}\cdot o\cdot {i}_{{{{\rm{CaL}}}}},$$

where *A* is the area of the cell membrane (in *μ*m^2^), *C*_*m*_ is the specific membrane capacitance (in pF/*μ*m^2^), *o* is the open probability (unitless), and *i*_CaL_ is the average current through a single open calcium channel (in pA). When a calcium channel blocker (CCB) is present, we assume that the current through a single calcium channel is reduced. We incorporate this in the model by introducing a scaling factor *b*(*D*) corresponding to the reduction in single-channel current

4 $${I}_{{{{\rm{CaL}}}}}=\frac{n{N}_{0}}{A{C}_{m}}\cdot o\cdot b(D)\cdot {i}_{{{{\rm{CaL}}}}}.$$

When no drug is present, *b*(0) = 1, and when a drug blocks the current by, for instance, 90%, then *b*(*D*) = 0.1. A description of how the model from refs. ^4^,^8^ can be rewritten to the form (1) and (2) is provided in the Supplementary Information (Supplementary Note 1).

**Fig. 1:**
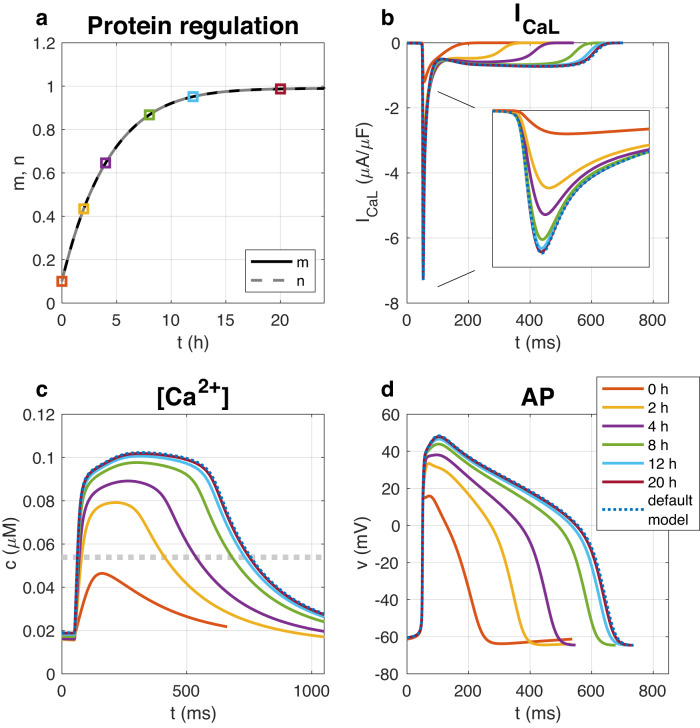
Simulation of the system (1) and (2) included in an AP model for hiPSC-CMs. We use *τ*_*m*_ = 400 mMms and *τ*_*n*_ = 1000 ms and start the simulation from a starting point of *n* = *m* = 0.1. **a** Shows the time evolution of *m* and *n*. In addition, six time points are marked. In the next three figure panels, the calcium current density over the cell membrane, *I*_CaL_ (**b**), the cytosolic calcium concentration, *c* (**c**), and the action potential (AP) (**d**) are plotted at these six points in time. In addition, we show the default model solution, and we observe that after 20 h of simulation, *m* and *n* are very close to 1 and the model solution is very close to the default model solution. The dotted gray line in the lower left plot shows the target calcium concentration, c*.

In Fig. 1, the properties of this model are demonstrated in conjunction with a mathematical model of the action potential for hiPSC-CMs (see ref. 10 and Supplementary Note 6). Initially, we set *n* = *m* = 0.1, and the initial conditions for the remaining model variables (including *c*) are found by running a simulation of the model with *n* fixed at 0.1. The parameter c* is set as the average cytosolic calcium concentration during an action potential (AP) cycle in the default model with *n* = 1. Furthermore, we set *τ*_*m*_ = 400 mMms and *τ*_*n*_ = 1000 ms. The model forecasts a significant upregulation in the number of calcium channels, leading to a normalization of the intracellular calcium concentration. After a span of 20 h, the ion channel count is fully restored, and the intracellular calcium concentration, *c*, attains its target level, c*, on average during an AP cycle. Notably, this membrane rectification process unfolds over hours, contrasting with the brief duration of each action potential, which lasts between 200–600 ms. This example underscores the model’s utility: if the intracellular calcium concentration diverges from the target level, the ion channel count will be modulated to bring the concentration back to equilibrium. This aligns with the model’s original intent, as corroborated by studies such as refs. ^3,4^.

Calcium channel blockers (CCBs) are a class of drugs that have wide clinical cardiovascular applicability, including the treatment of hypertension, angina, and cardiac arrhythmias^11^. Through L-type calcium antagonism, they can have an effect on both vascular smooth muscle and myocardial muscle, and can cause the reduction of blood pressure, coronary-artery dilation, and depression of cardiac contractility^12^. In the heart, the block of *I*_CaL_ diminishes the influx of calcium into the intracellular space during an AP. This, in turn, results in a decreased release of calcium from internal storage units, because of the effect commonly known as graded release (see, e.g., refs. 13–15). Consequently, the average intracellular calcium concentration should be reduced when exposed to CCBs. In the presented framework, when the intracellular calcium concentration *c* falls below the target value c*, the model predicts an upregulation in the expression of calcium channels, thereby increasing the calcium influx into the cell. If this trend continues unchecked, the inhibitory effect of the CCB may therefore be nullified.

**Fig. 2:**
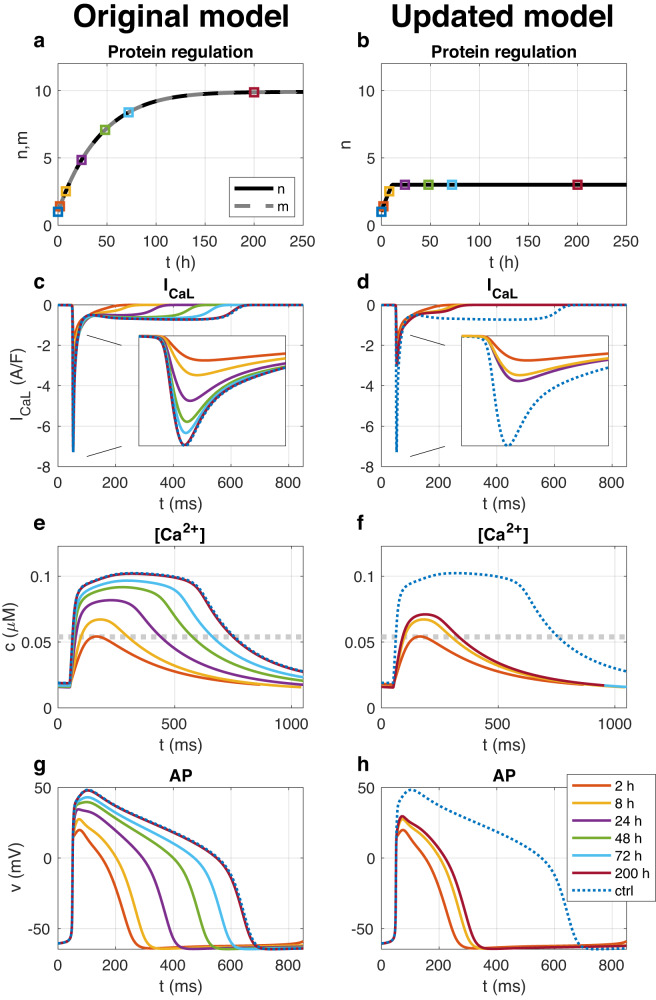
Simulation of calcium channel block in the original model (1) and (2) and in the updated model (13). Left panels: Simulation of the system (1) and (2) included in an AP model for hiPSC-CMs with *τ*_*m*_ = 400 mMms and *τ*_*n*_ = 1000 ms from a starting point of *n* = *m* = 1 and a 90% block of *I*_CaL_ (*b*(*D*) = 0.1). Initially, *c* is considerably reduced by the *I*_CaL_ block, leading to an increase in *m* and *n*. Eventually, *m* and *n* reach a value of 10, restoring *I*_CaL_ to its default strength as well as restoring the cytosolic calcium concentration and the AP to the default (control, ctrl) case. Right panels: Simulation of the same case using the updated model (13) with *τ*_*n*_ = 400 mMms, *n*_−_ = 0.1 and *n*_+_ = 3. In this case, *n* cannot increase above *n*_+_, and the model reaches an equilibrium solution at *n* ≈ *n*_+_ and the blocking of *I*_CaL_ is not completely diminished over time. **a**, **b** Show the time evolution of *m* and *n*, and the remaining panels show the calcium current density, *I*_CaL_ (**c** and **d**), the cytosolic calcium concentration (**e** and **f**), and the membrane potential (**g** and **h**) during an AP at six different time points after the *I*_CaL_ blocking is applied, as well as in the control case.

An illustration of the model is provided in the left panel of Fig. 2. In this simulation, the calcium current is reduced by 90% (i.e., *b*(*D*) = 0.1 in (3)). As the blocking lowers the average cytosolic calcium concentration, the model anticipates an increase in the number of ion channels, eventually counteracting the blocking effect; the calcium target is met, and the current strength is completely restored.

This example highlights the need for model refinement when considering the presence of CCBs; unlimited growth in the number of ion channels is not realistic. In the following sections, we demonstrate that the model can be simplified to a scalar equation, imposing a limit on ion channel growth. Additionally, we show that the model’s predictions align well with experimental observations using hiPSC-CMs. In summary, both the mathematical model and the in vitro measurements indicate a time-dependent effect of the CCB. The blocking effect is most pronounced in the initial hours but gradually diminishes, without being completely nullified, consistent with the theory that channel expression will increase when calcium concentrations fall below the target level.

## Results

We will address the effect of CCBs using a mathematical model motivated by the system (1) and (2). However, since *m* and *n* are very similar, we will, in the following sections, demonstrate that the original model system of ordinary differential equations can be reduced from a system of two equations to a scalar equation. In addition, we will impose a limit on channel growth which will be justified below. Furthermore, we will illustrate that the updated model coupled to a model for the action potential of hiPSC-CMs is able to qualitatively represent the temporal changes observed in the effect of the CCB nifedipine on a collection of hiPSC-CMs.

### Reduction to a scalar model

Both in Fig. 1 and in Fig. 2 we noticed that *m* ≈ *n*. This motivates analysis of the deviation given by

5 $$p=| m-n| .$$

From the system (1) and (2) we get,

6 $${p}^{{\prime} }={{{\rm{sign}}}}(m-n)({m}^{{\prime} }-{n}^{{\prime} })$$

7 $$={{{\rm{sign}}}}(m-n)\left(\frac{1}{{\tau }_{m}}({c}^{* }-c)-\frac{1}{{\tau }_{n}}(m-n)\right)$$

8 $$\le \frac{1}{{\tau }_{m}}| {c}^{* }-c| -\frac{1}{{\tau }_{n}}| m-n|$$

9 $$\le \frac{1}{{\tau }_{m}}| {c}^{* }-c{| }_{\infty }-\frac{1}{{\tau }_{n}}p,$$

where ∣c* − *c*∣_*∞*_ denotes the largest deviation of *c* from the target value c*. From Gronwall’s inequality (ref. 16, p. 283) we get

10 $$p(t)\le {e}^{-t/{\tau }_{n}}\left(p(0)-\frac{{\tau }_{n}}{{\tau }_{m}}| {c}^{* }-c{| }_{\infty }\right)+\frac{{\tau }_{n}}{{\tau }_{m}}| {c}^{* }-c{| }_{\infty }.$$

Since *τ*_*n*_ is on the order of a few seconds or less, we see that for large values of *t* (hours), we have

11 $$p\,\lesssim\, \frac{{\tau }_{n}}{{\tau }_{m}}| {c}^{* }-c{| }_{\infty }.$$

Numerical experiments reveal that in order to fit data from measurements of hiPSC-CMs (see below), we need the parameter *τ*_*m*_ to be around 400 mM × ms. Furthermore, varying *τ*_*n*_ between 10 ms and 10,000 ms did not seem to influence the main results (see the Supplementary Note 2). Moreover, we observe that ∣c* − *c*∣_*∞*_ never exceeds 5 × 10^−5^ mM. Therefore, we have

12 $$p=| m-n\left[\,\lesssim\, \right.1{0}^{-3},$$

so *m* ≈ *n* and we can reduce the system to a scalar equation in *n*.

### Introducing upper and lower bounds on the protein expression

We have seen that the original model runs into difficulties when the calcium current is blocked. The source of the difficulty is that the number of ion channels are allowed to grow in an unlimited manner. This is not realistic, and we want to adjust the model accordingly by putting an upper limit, *n* = *n*_+_, on how much the number of calcium ion channels can grow. In addition, we wish to enforce a lower limit, *n* = *n*_−_, so that the number of ion channels cannot become negative. To this end, we introduce the model

13 $${\tau }_{n}\frac{dn}{dt}=({c}^{* }-c)H(c,n),$$

14 $$H(c,n)=h(n,{n}_{-},{\varepsilon }_{n})h(c,{c}^{* },{\varepsilon }_{c})+h({n}_{+},n,{\varepsilon }_{n})h({c}^{* },c,{\varepsilon }_{c}),$$

15 $$h(a,b,\varepsilon )=\frac{1}{2}\left(1+\tanh \left(\frac{a-b}{\varepsilon }\right)\right).$$

**Fig. 3:**
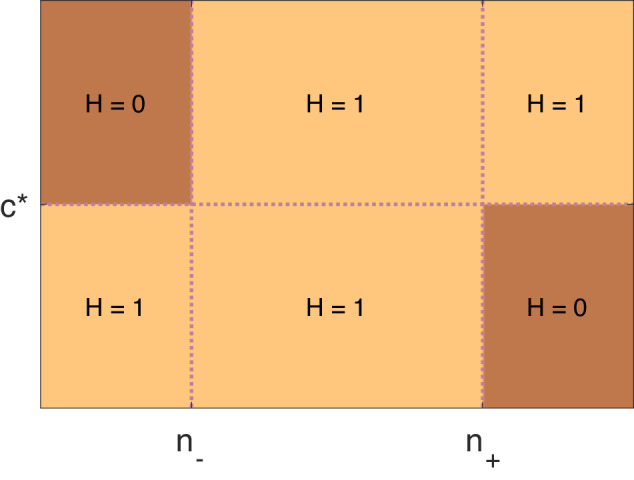
Illustration of the function *H* defined in (14) and (15). The function is 1 everywhere except that it reduces to 0 when *n* < *n*_−_ and *c* > c* and when *n* > *n*^+^ and *c* < c*. The steepness of the transition between the different values of *H* is controlled by the *ε*_*n*_ and *ε*_*c*_ parameters.

Here, the parameters *τ*_*n*_, *n*_−_, *n*_+_, *ε*_*n*_ and *ε*_*c*_ must be estimated. In the computation, we have used *n*_−_ = 0.1, *n*_+_ = 3, *τ*_*n*_ = 400 mMms, *ε*_*n*_ = 0.01 and *ε*_*c*_ = 10^−7^ mM. These values were based on hand-tuning in order to fit the experimental data. Moreover, c* is the average cytosolic calcium concentration during an AP cycle in the default model with *n* = 1. Note that *n* is a dimensionless number and *nN*_0_ is the total number of ion channels in the membrane. The unit of concentrations are mM and time is in ms, hence the unit of *τ*_*n*_ is ms × mM. The function *H* is introduced to bound the number of ion channels and is illustrated in Fig. 3.

The model (1) and (2) reaches its equilibrium when the calcium concentration reaches the target value, i.e., when *c* ≈ c*. The new model (13) can reach an equilibrium state either if the target value is achieved, if the maximum number of calcium ion channels is reached, i.e., if *n* ≈ *n*_+_, or if the minimum number of calcium ion channels is reached, i.e., if *n* ≈ *n*_−_.

In the right panel of Fig. 2, we have rerun the *I*_CaL_ block example illustrated in the left panel of Fig. 2 using the updated version of the model (i.e., (13)). In the right panel of Fig. 2, we observe that in the new model, the number of calcium channels, *n*, initially increases in response to the reduced calcium concentration resulting from the reduction of *I*_CaL_. However, when *n* reaches the value of *n* = *n*_+_ = 3, *n* is not able to increase further and reaches an equilibrium solution even though the average cytosolic calcium concentration has not reached the target concentration, c*. In other words, the effect of the *I*_CaL_ block is reduced over the initial hours after *I*_CaL_ block, but *I*_CaL_ is not able to fully recover as in the left panel for the original model (1) and (2).

### Simulation and measurements of the effect of nifedipine on hiPSC-CMs

We will now show that the updated model described above is able to qualitatively represent the temporal changes observed in the effect of the CCB nifedipine on a collection of hiPSC-CMs. We consider two doses of nifedipine, 0.1 μ M and 1 μM. The effect of 0.1 μM of nifedipine was modeled by reducing *I*_CaL_ by 55% whereas the effect of 1 μM of nifedipine was modeled by reducing *I*_CaL_ by 88%. In other words, *b*(0.1 μM) = 0.45 and *b*(1 μM) = 0.12 in (3). These blocking percentages are in relatively good agreement with measurements of the drug effect from literature (see Supplementary Note 3).

#### Effect of 0.1 μM of nifedipine

**Fig. 4:**
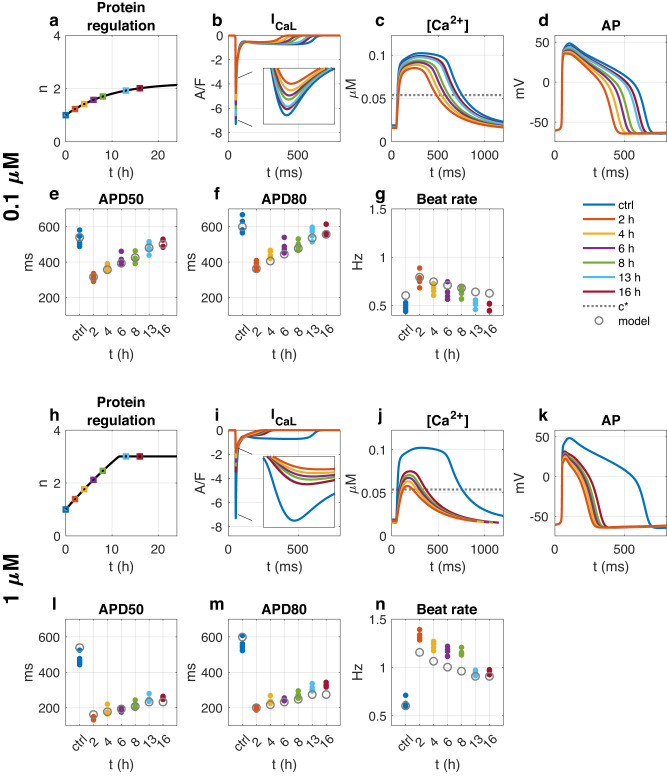
Simulated and measured effect of nifedipine on spontaneously beating hiPSC-CMs. **a**–**g** Show the effect of 0.1 μM of nifedipine and **h**–**n** show the effect of 1 μM of nifedipine. **a**–**d** and **h**–**k** show properties of the model solution for the 0.1 μM and 1 μM doses, respectively. More specifically, **a** and **h** show the time evolution of the number of calcium channels, *n*. The next plots show *I*_CaL_ (**b** and **i**), the cytosolic calcium concentration (**c** and **j**) and the action potential (**d** and **k**) measured in the control case (ctrl, no *I*_CaL_ block) and at six time points after the *I*_CaL_ block was applied. Furthermore, **e**–**g** and **l**–**n** show the APD50 (**e** and **l**), APD80 (**f** and **m**) and beat rate (**g** and **n**) sampled in the control case and at the six points in time. The open gray circles are computed from the model solution and the filled colored circles are measurements of hiPSC-CMs in the control case (blue) and after exposure to nifedipine. In the model, 0.1 μM and 1 μM of nifedipine were represented by reducing *I*_CaL_ by 55% and 88%, respectively, i.e. *b*(0.1 μM) = 0.45 and *b*(1 μM) = 0.12 in (3).

Figure 4 shows the results of simulations of the two doses of nifedipine as well as biomarker values collected from measurements of hiPSC-CMs. In Fig. 4a–g, 0.1 μM of nifedipine is applied. In the simulation results reported in Fig. 4a–d, we observe that the reduction of *I*_CaL_ cause a reduced cytosolic calcium concentration, which leads to a gradual increase in the number of calcium channels, *n*. Furthermore, after 16 h of drug exposure, the number of calcium channels has increased so much that *I*_CaL_, the calcium concentration and the AP is almost completely recovered to the control case. In Fig. 4e–g, three biomarkers are computed in the control case before drug exposure and after 2 h, 4 h, 6 h, 8 h, 13 h and 16 h of drug exposure. The open gray circles are computed from the model solution and the filled colored circles are computed from optical measurements of hiPSC-CMs. For both the model and in the measurements of hiPSC-CMs, we clearly observe that the initial decrease in APD and increase in beat rate gradually is reduced over the 16 h of drug exposure. Furthermore, both the measured data and the model results suggest that the drug effect has almost completely diminished after 16 h.

#### Effect of 1 μM of nifedipine

In Fig. 4h–n, 1 μM of nifedipine is applied. Again, we observe an initial decrease in cytosolic calcium concentration resulting in a gradual increase in the number of calcium channels, *n*. However, the maximum number of calcium channels, *n*_+_ is in this case reached before *I*_CaL_ is fully recovered and the increase in *n* halts. Consequently, even after 16 h of drug exposure the effect of the drug is still considerable, although somewhat less pronounced than after only 2 h of drug exposure.

### Investigating the effect of adjusting the number of several protein types

**Fig. 5:**
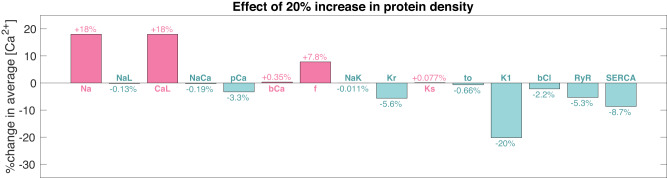
Effect on the cytosolic calcium concentration of adjusting the number of different types of proteins (channels, pumps and exchangers) in the membrane model without a dynamic number of calcium channels. The figure shows the percent change in the average cytosolic calcium concentration resulting from a 20% increase in the number of each of the types of channels, pumps and exchangers in the model. The effects are measured by comparing the average cytosolic calcium concentration over 10 s of simulation, 1000 s after the parameter change was applied, and comparing it to the average (over 10 s) in a simulation of the default model.

In the original models from refs. 4 and 8, the cytosolic calcium concentration is not only assumed to influence the number of calcium channels in the cell membrane, it is assumed to influence the number of all (or most of) the channels, pumps and exchangers considered in the models. In Fig. 5, we investigate the effect on the cytosolic calcium concentration of adjusting several protein types in the hiPSC-CM base model. The figure reports the effect of increasing each protein type separately and keeping the number of the remaining protein types constant. We observe that increasing the number of sodium (Na) or funny current (f) channels both have the similar effects on the calcium concentration as increasing the number of L-type calcium channels. That is, increasing the number of channels increases the cytosolic calcium concentration. However, increasing the number of, e.g., Kr or K1 potassium channels or the number of SERCA pumps, has the opposite effect. That is, increasing the number of these types of proteins, decreases the intracellular calcium concentration.

## Discussion

A series of recent papers propose that the gene expression of membrane proteins in excitable cells is regulated by the intracellular calcium concentration, *c*, relative to a target concentration, c*, see, e.g., refs. 3,4,8. Simply put, if *c* < c*, the cell compensates by increasing the number of ion channels, aiming to elevate *c* to c*. This hypothesis has been formulated through ordinary differential equations, which can be integrated into existing mathematical models of cellular action potentials.

Here, we consider the implications of this theory when a calcium channel blocker (CCB) is introduced in a model where the number of L-type calcium channels is governed by such a mechanism. The application of a CCB leads to a reduction in calcium influx, resulting in *c* < c*. According to the theory, this will trigger an increase in the number of calcium ion channels. While each individual channel’s blocking by the CCB remains constant, the overall cellular calcium current would increase due to the greater number of channels, changing the CCB’s effect. The theory suggests this process would continue until *c* = c*, a notion we find implausible given the well-documented efficacy of CCBs in reducing calcium currents. To address this, we have refined the mathematical model by imposing limits on the minimum and maximum number of ion channels.

Our updated model aligns reasonably well with empirical data as illustrated in Fig. 4. The model predicts that the CCB’s impact is most pronounced immediately after administration, gradually diminishing over subsequent hours. The implemented model modifications ensure that the effect of the CCB does not completely disappear, but instead stabilizes at a significant level, in alignment with experimental observations.

Our findings suggest that the efficacy of CCBs on whole-cell currents of cardiac cells should be evaluated over an extended timeframe, rather than solely immediately post-administration. This is as dynamic changes to the calcium control system may confound correct interpretation of drug effects. We do not anticipate any temporal variations in the efficacy of CCBs at the single-channel level; the observed temporal effects are only attributed to changes in gene expression induced by changes in intracellular calcium concentrations.

Previous studies (refs. 4 and 8) have suggested that intracellular calcium concentrations influence not only the calcium current, but also other membrane currents and intracellular calcium storage systems. We have concentrated on the L-type calcium current to elucidate its role in experimental observations, consciously omitting the impact of other membrane current variations. This is based on three considerations:

- *Simplicity in parameterization:*
- In general, we aim at limiting the number of adjustable parameters.
- *Complex interactions with other currents:*
- The effect of perturbing currents and fluxes on the intracellular calcium concentration are presented in the Fig. 5. We note that increase in the L-type calcium current (*I*_CaL_) results in increased calcium concentration. Similarly, increased sodium current (*I*_Na_) also increases the intracellular calcium concentration. Therefore, these two currents may be regulated by the same mechanism aiming at reaching the calcium target level. However, increasing the potassium currents lead to a reduced intracellular calcium concentration. Therefore, similar regulation of all membrane currents is not straightforward for the model of the hiPSC-CMs considered here.
  In the Supplementary Information (Supplementary Note 5) we have have also reported experiments using other action potential models in order to examine the effect of perturbing various currents on the intracellular calcium concentrations. Specifically, we have considered the Paci et al. model^17^ and the Kernik et al. model^18^ for hiPSC-CMs, and the Severi et al. model^19^ for sinoatrial node cells. For all models, we observe that increasing the calcium current leads to increased intracellular calcium concentration, whereas at least one of the other currents work the other way around.
- *Specificity of nifedipine effects:*
- If the intracellular calcium concentration regulates the density of all other membrane currents, then the application of nifedipine should significantly influence these currents alongside the calcium current. However, nifedipine is commonly only identified as a calcium channel blocker (see, e.g., ref. 20). On the other hand; the effects caused by protein regulation are quite slow (many hours), and it not clear whether the effect of nifedipine on other currents have been measured hours after application of the drug. Also, the effect of protein regulation would be observable only in whole cell measurements and not in single channel experiments.

As pointed out in ref. 21, the regulatory mechanism of gene expressions is not generally understood and can be based on several different strategies: a) channel genes could be regulated independently, b) channels could be regulated coordinately according to cell size maintaining an appropriate ratio of channel types, c) channels could be regulated in order to achieve acceptable cell activity, or d) a blend of these mechanisms. In conclusion, we recognize that perturbations to the intracellular calcium concentration may induce concurrent changes in other channels and regulatory mechanisms. However, we believe it is judicious to refrain from expanding our model until data suggest otherwise.

Calcium channel blockers (CCBs) are widely used in clinical practice, as evidenced by multiple studies, see, e.g., refs. 22,23. Given the well-characterized, long-term applications of CCBs in clinical settings, our findings are unlikely to cause changes in clinical practice. However, it is worth noting that the initial potency of CCBs may significantly exceed their long-term effects, a nuance that could be relevant for future research and treatment strategies. It is also worth noting that if calcium homeostasis mechanisms can alter the transcriptome of key ion channels, this phenomena may have an important consideration in processes that depend on the delicate balance of membrane ion channels, such as the generation of cardiac arrhythmias.

While our study employs mathematical models to analyze gene expression changes and utilizes calcium-sensitive dyes to measure the effects of CCBs on hiPSC-CMs, other researchers have directly measured the cardiomyocyte transcriptome following CCB treatment. In ref. 24, it was observed that *"CCB can lose its inhibitory effect on L-type calcium channels after chronic treatment in some iPSC-CM lines."* Furthermore, the authors add that *"While acute treatment of CCBs exerted expected negative chronotropic and inotropic effects in all lines, we observed line-specific recovery after long-term treatment in two lines. Electrophysiological study showed that the calcium current was no longer inhibited in those lines after chronic treatment."* It should be noted that changes in gene expression is a complex process and the modeling framework considered here is therefore a coarse representation of this process. In sum, the combined results motivates further analysis of time-dependent effect of CCB and other drugs affecting the membrane of cardiomyocytes.

To sum up, we have used a recently developed mathematical model to clarify the effects of the calcium channel blocker (CCB) nifedipine. This model is designed to simulate the dynamics of ion channel density, specifically for those channels facilitating calcium ion transport, with the aim of maintaining a target value of the mean intracellular calcium concentration. Our primary goal was to understand the repercussions of this regulatory mechanism when the intracellular calcium concentration is altered due to the introduction of a CCB. Experimental observations using human induced pluripotent stem cell-derived cardiomyocytes (hiPSC-CMs) demonstrate a time-dependent effectiveness of nifedipine. Our study confirms that these experimental results align with the predictions of the mathematical model. We conclude that the time-dependent efficacy of nifedipine may be attributed to its influence on the density of calcium-carrying ion channels.

## Methods

### Numerical methods

In all our numerical simulations, we use the ODE solver *ode15s* in MATLAB.

### Measuring the effect of CCB in hiPSC-CMs

Cardiac microtissues were generated based on a refined version of our previously published cardiac microtissue platform^25,26^. In brief, microtissues were created in 384 well plates (206384; GraceBioLabs) with custom substrates (COC TPE E140; Stratec) featuring 4 tissue formation chambers per well (1400 × 200 × 150 μm length × width × depth) using 35,000 iPSC-derived cardiomyocytes (C1056; Fujifilm CDI) per well. Microtissues were used at day 29 and stained with 500 nM voltage sensitive dye (PhoS1; Photoswitch Biosciences). Nifedipine (PHR1290; Sigma) was freshly prepared as 6-fold concentrate in cell culture media (M1003; Fujifilm CDI) from a 10 mM stock in DMSO. After baseline recordings, 10 μL of concentrated drug were added to 50 μL culture media in each well to achieve the desired dose of 0.1 μM or 1 μM. Repeated recordings were performed at 2 h, 4 h, 6 h, 8 h, 13 h and 16 h following drug addition. All imaging was performed inside the incubation chamber of an ImageXpress Micro (Molecular Devices) microscope. Cy-5 fluorescence (1.5% laser power) was recorded in each well for 8 s at 50 fps via a 4x objective with 2 × 2 binning. Each video was then segmented to obtain voltage traces for individual tissues and biomarkers (APD50, APD80 and beat rate) were computed. The approach used to compute the biomarkers is described in Supplementary Note 4. There, the recorded experimental traces are also shown.

### Ethics statement

All experimental work was performed using Fujifilm Cellular Dynamics commercially available and licensed iCell cardiomyocytes, derived from hiPSC cells obtained with donor consent. All work was approved under a Biological Use Authorization from the University of California, Berkeley.

### Disclosure of writing assistance

During the preparation of this manuscript, the authors utilized the ChatGPT4 language model to enhance the language quality for contributions from non-native English speakers. Subsequent to this automated assistance, the authors rigorously reviewed and edited the manuscript to ensure its accuracy and integrity. The authors assume full responsibility for the content of the publication.

### Reporting summary

Further information on research design is available in the Nature Research Reporting Summary linked to this article.

### Supplementary information

Supplementary Information

Reporting Summary

## Data Availability

The data generated in this study are publicly available at Zenodo: 10.5281/zenodo.10245940^27^.

## Code availability

The code generated in this study are publicly available at Zenodo: 10.5281/zenodo.10245940^27^.
