# Supplementary material for: Do calcium channel blockers applied to cardiomyocytes cause increased channel expression resulting in reduced efficacy?
Source: NPJ Syst Biol Appl. 2024 Mar 1;10:22. doi: 10.1038/s41540-024-00347-3 (PMC10907638; doi:10.1038/s41540-024-00347-3)
Supplement: Supplementary file 1 — Supplementary Information [file 41540_2024_347_MOESM1_ESM.pdf]

Supplementary Information for 'Do calcium channel blockers applied to cardiomyocytes cause increased channel expression resulting in reduced efficacy?'

Supplementary Note 1: Reformulating the protein regulation model from [1, 2]

In [1, 2], the protein regulation model is given in the form[1]

$$\tau_r \frac{dr}{dt} = c^* - c, \tag{1}$$

$$\tau_g \frac{dg}{dt} = r - g. \tag{2}$$

Here, c is the cytosolic calcium concentration, c^* is the target calcium concentration, g is the conductance of the considered type of ion channel, r is a intermediary variable, and τ_r and τ_g are time constants.

In our computations, we have rewritten the original formulation (1)–(2) to instead of r and g, model unitless scaling factors n and m for the number of ion channels in the cell membrane and the number of messenger RNAs (mRNAs). More specifically, the number of ion channels and the number of mRNAs are given by $N(t) = n(t)N_0$ and $M(t) = m(t)M_0$, respectively, where N_0 and M_0 are the default number of channels and mRNAs.

The total current through all the I_{CaL} channels in the cell membrane can be expressed as

$$I_{\mathrm{CaL}}^{\mathrm{tot}} = n \cdot N_0 \cdot g_0 \cdot o \cdot i_{\mathrm{CaL},0}, \tag{3}$$

[1] Note that we here use the notation r for the variable that is called m in [1, 2]. The reason is to avoid confusion with the variable m in the formulation of the model used in this study.

where g_0 is the single channel conductance of the channel, o is the open probability of the calcium channels and $i_{\mathrm{CaL},0}$ is an expression for how the single-channel current depends on model variables like the membrane potential and the cytosolic calcium concentration. In this setting, the conductance g in the model (1)–(2) is given by

$$g = n \cdot N_0 \cdot g_0 = n \cdot \bar{g}, \tag{4}$$

where $\bar{g} = N_0 \cdot g_0$ is the default value of g (corresponding to $n = 1$). In other words, we have

$$n = \frac{g}{\bar{g}} \tag{5}$$

In order to rewrite (1)–(2) to a system for n and m, we similarly define

$$m = \frac{r}{\bar{g}} \tag{6}$$

and divide both (1) and (2) by $\bar{g}$. This yields

$$\tau_r \frac{d\left(\frac{r}{\bar{g}}\right)}{dt} = \frac{1}{\bar{g}}\left(c^* - c\right), \tag{7}$$

$$\tau_g \frac{d\left(\frac{g}{\bar{g}}\right)}{dt} = \frac{r}{\bar{g}} - \frac{g}{\bar{g}}, \tag{8}$$

which can be rewritten as

$$\tau_m \frac{dm}{dt} = c^* - c, \tag{9}$$

$$\tau_n \frac{dn}{dt} = m - n, \tag{10}$$

where

$$\tau_m = \tau_r \cdot \bar{g}, \tag{11}$$

$$\tau_n = \tau_g. \tag{12}$$

Supplementary Note 2: Investigating the effect of adjusting τ_n

In Supplementary Figure 1, we show the time evolution of m and n in a simulation with the model (9)–(10) coupled to the action potential model of hiPSC-CMs described in Supplementary Note 6. The simulation is started from $n = m = 0.1$ in exactly the same manner as in Figure 1 in the paper. We let $\tau_m = 400$ mMms (like in the paper) and vary τ_n between 100 ms and 10,000 ms. For comparison, the value $\tau_n = 1000$ ms was used in [2]. We observe that all the considered values of τ_n provide almost identical solutions.

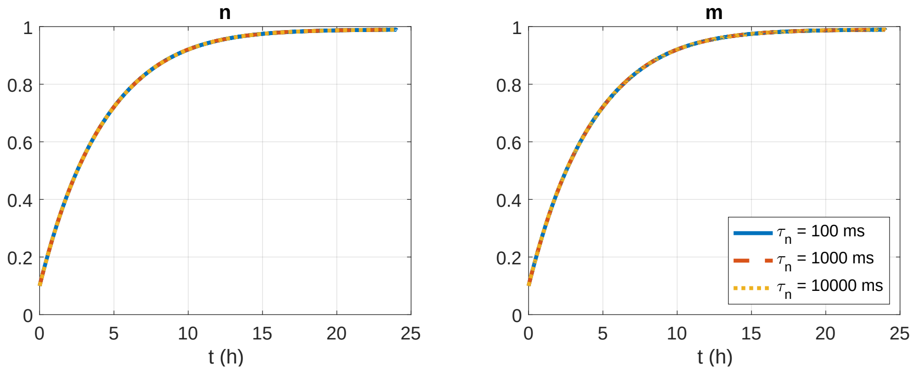

Supplementary Figure 1: Time evolution of the solutions n and m of the model (9)–(10) coupled to an action potential model of hiPSC-CMs for different values of τ_m. The simulations are started from $n = m = 0.1$, and we use $\tau_m = 400$ mMms.

Supplementary Note 3: Comparison of nifedipine block percentages from literature

In order to compare the assumed block percentages associated with 0.1 μM and 1 μM of nifedipine, we identified dose-dependent effects on I_{CaL} from literature. The selected block percentages are compared to the data from literature in Supplementary Figure 2. The figure shows the value of $b(D)$ as a function of the dose, D, reported in different studies in addition to the values used in this study. More specifically, the effect of the drug is typically reported in terms on a half-maximal effective concentration, EC_{50}, a Hill coefficient, h and a maximal effect E, and $b(D)$ is assumed to be given by

$$b(D) = 1 + \frac{D^h E}{D^h + (\text{EC}_{50})^h}. \tag{13}$$

Here, as an example, $E = -1$ if the maximal effect of the drug is to block the current completely, and $E = -0.5$ if the maximal effect is to block the current by 50%. The values of EC_{50}, h, and E used to generate the curves in Supplementary Figure 2 are reported in Supplementary Table 1. In cases where h or E are not provided, we have assumed $h = 1$ and $E = -1$.

Reference	EC_{50}	h	E
Kramer et al. 2013 [6]	0.012 μM	1.02	-0.883
Romero et al. 2018 [7]	0.052 μM		
DiStilo et al. 1988 [8]	0.060 μM		
Gibson et al. 2014 [9]	0.039 μM	0.85	
Ma et al. 2011 [10]	0.038 μM		

Supplementary Table 1: Dose-dependent effects of nifedipine on the L-type calcium current from literature used in Supplementary Figure 2. The blocking effect is modeled using (13).

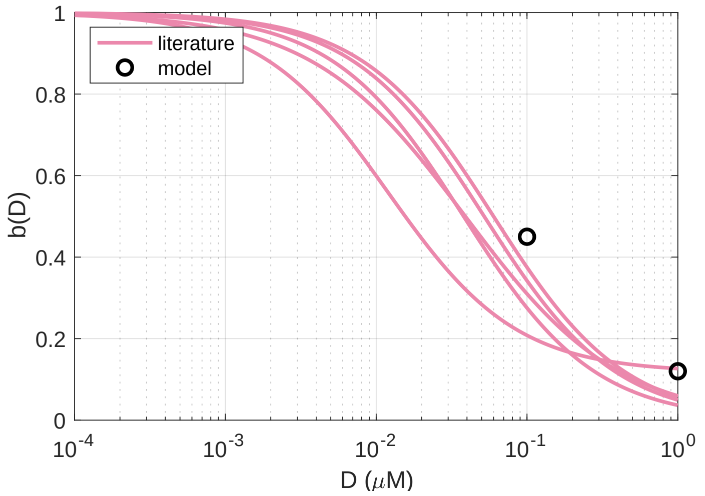

Supplementary Figure 2: Comparison of the blocking effect of nifedipine $b(D)$ used in our model to data from literature.

Supplementary Note 4: Experimental traces

In Supplementary Figures 3 and 4, we report the experimental voltage traces used to compute the biomarkers reported in Figure 4 in the paper. We show the baseline traces as well as the traces recorded 2 h, 4 h, 6 h, 8 h, 13 h and 16 h after application of nifedipine. For the dose of 0.1 μM of nifedipine, we have data from five different tissues of hiPSC-CMs, and for 1 μM, we have data from seven tissues. The traces are recorded using voltage sensitive dyes, and we consider the average fluorescence (spatially) over each tissue. The APD50 and APD80 values reported in Figure 4 in the paper are computed using the representative single AP traces marked in orange in the plots and the beat rate is computed using the average of all the recorded beats. The representative single AP traces are selected as the AP with the smallest total difference to the median (temporally) of the biomarkers computed for each of the APs in the trace. In Figure 4 in the paper, the APD and beat rate values are represented by a single dot for each of the considered tissues.

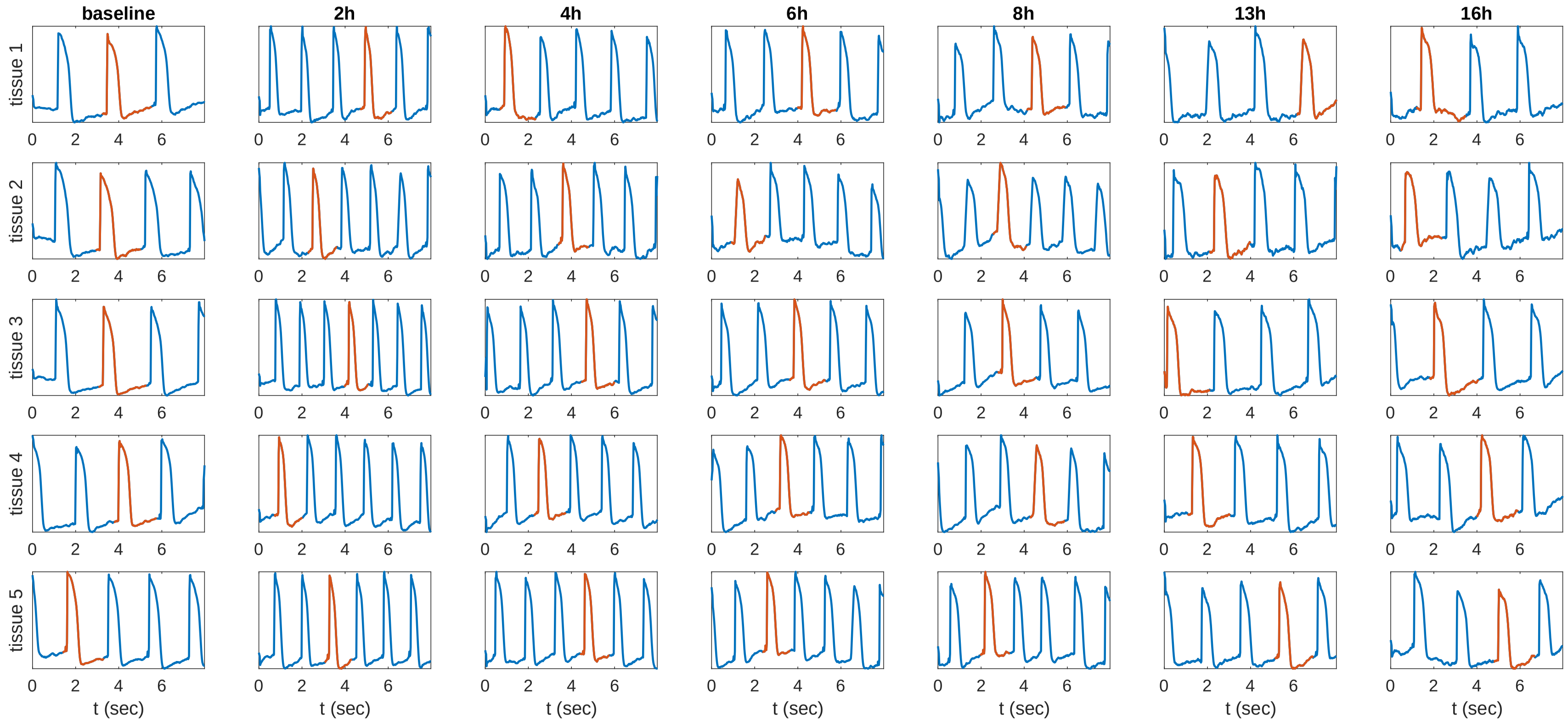

Supplementary Figure 3: Experimental voltage traces recorded using voltage sensitive dyes for baseline and after 2 h, 4 h, 6 h, 8 h, 13 h, and 16 h application of 0.1 μM of nifedipine. The APD50 and APD80 values reported in Figure 4 in the paper are computed using the representative traces marked in orange and the beat rate is computed from all the recorded beats.

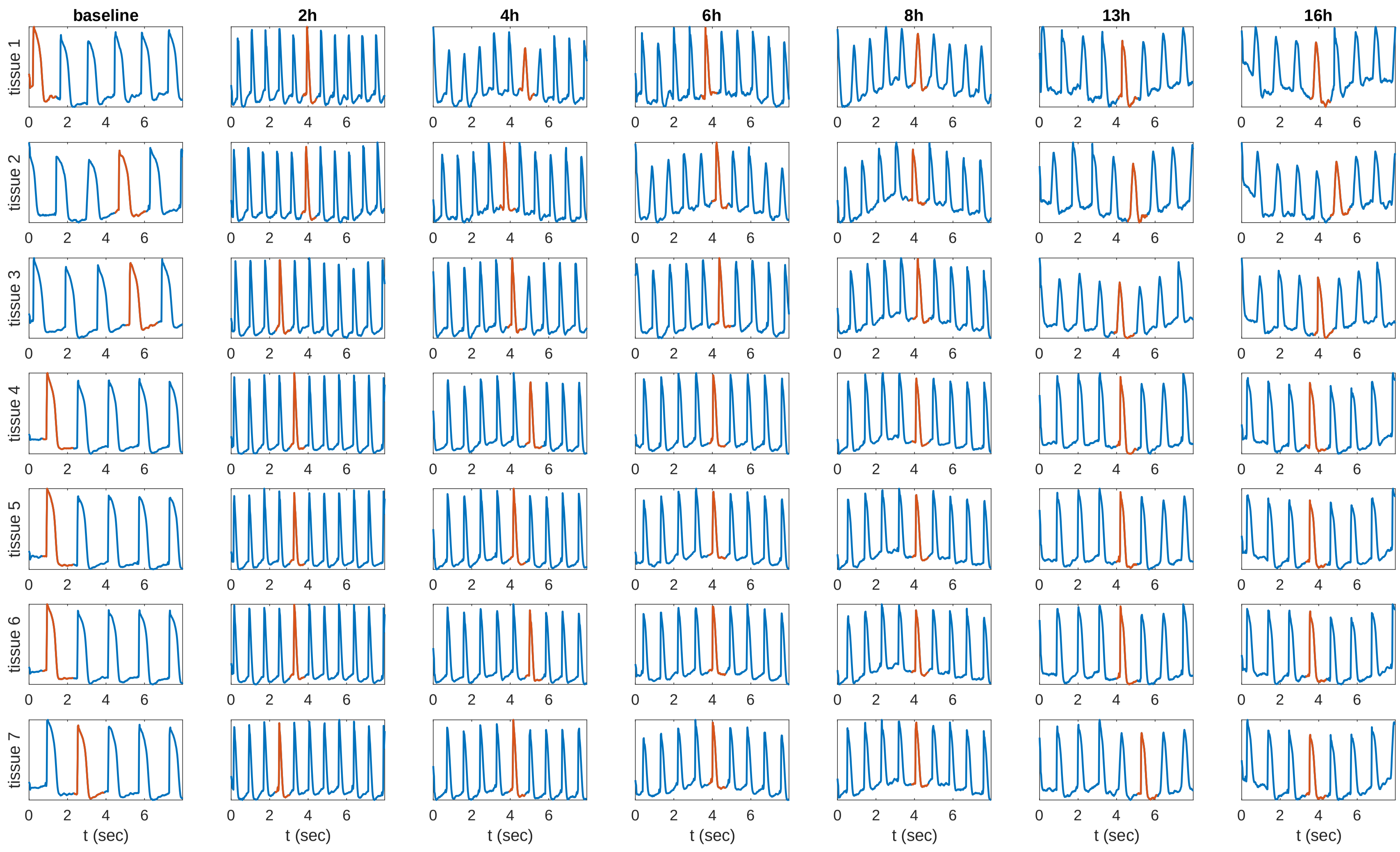

Supplementary Figure 4: Experimental voltage traces recorded using voltage sensitive dyes for baseline and after 2 h, 4 h, 6 h, 8 h, 13 h, and 16 h application of 1 μM of nifedipine. The APD50 and APD80 values reported in Figure 4 in the paper are computed using the representative traces marked in orange and the beat rate is computed from all the recorded beats.

Supplementary Note 5: Investigating the effect of adjusting the number of different protein types in other membrane models

In Supplementary Figure 5, we investigate the effect of increasing the number of different types of proteins in three other membrane models. More specifically, we consider the hiPSC-CM models of Paci et al. 2013 [3] and Kernik et al. 2019 [4]. In addition, we consider the sinoatrial node (SAN) model of Severi et al. 2012 [5], which is the model used in [2]. We observe that for all the considered models, the intracellular calcium concentration increases when the number of some of the protein types increases and that the intracellular calcium concentration increases decreases when the number of other protein types increases.

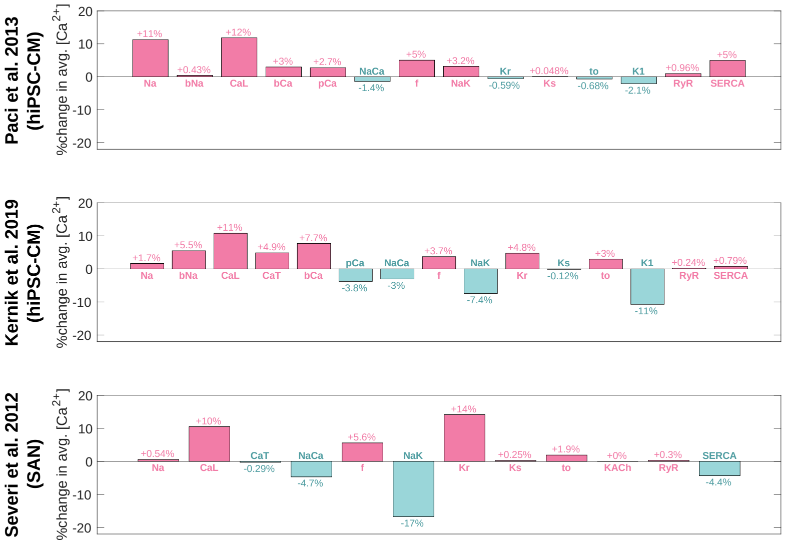

Supplementary Figure 5: Investigation of the effect on the cytosolic calcium concentration of adjusting the number of different types of proteins (channels, pumps and exchangers) in the other models. Ase report the percent change in the average cytosolic calcium concentration resulting from a 20% increase in the number of each of the types of channels, pumps and exchangers in the model. The effects are measured by comparing the average cytosolic calcium concentration over 10 seconds of simulation, 2 minutes after the parameter change was applied, and comparing it to the average (over 10 seconds) in a simulation of the default model.

Supplementary Note 6: Model formulation

In this note, the formulation of the base model for the action potential of hiPSC-CMs, based on [11, 12] is provided. In the model formulation, the membrane potential (v) is given in units of mV, the Ca^{2+} concentrations are given in units of mM, all currents are expressed in units of A/F, and the Ca^{2+} fluxes are expressed as mmol/ms per total cell volume (i.e., in units of mM/ms). The parameters of the model are given in Supplementary Tables 2–8.

Membrane potential

The membrane potential is governed by the equation

$$\begin{aligned} \frac{dv}{dt} = -(&I_{\mathrm{Na}} + I_{\mathrm{NaL}} + I_{\mathrm{CaL}} + I_{\mathrm{to}} + I_{\mathrm{Kr}} + I_{\mathrm{Ks}} + I_{\mathrm{K1}} \\ &+ I_{\mathrm{NaCa}} + I_{\mathrm{NaK}} + I_{\mathrm{pCa}} + I_{\mathrm{bCl}} + I_{\mathrm{bCa}} + I_{\mathrm{f}}), \end{aligned} \tag{14}$$

where I_{stim} is an applied stimulus current, and I_{Na}, I_{NaL}, I_{CaL}, I_{to}, I_{Kr}, I_{Ks}, I_{K1}, I_{NaCa}, I_{NaK}, I_{pCa}, I_{bCl}, I_{bCa}, and I_{f} are membrane currents specified below.

Membrane currents

The currents through the voltage-gated ion channels on the cell membrane are in general given on the form

$$I = go(v - E),$$

where g is the channel conductance, v is the membrane potential and E is the equilibrium potential of the channel. Furthermore, $o = \prod_i z_i$ is the open probability of the channels, where z_i are gating variables, either given as a function of the membrane potential or governed by equations of the form

$$z_i' = \frac{1}{\tau_{z_i}}(z_{i,\infty} - z_i). \tag{15}$$

The parameters τ_{z_i} and $z_{i,\infty}$ are specified for each of the gating variables of the model in Supplementary Table 9.

Fast sodium current The formulation of the fast sodium current is an adjusted version of the model given in [13], supporting slower upstroke velocities

more similar to those observed in the optical measurements of hiPSC-CMs. The current is given by

$$I_{\mathrm{Na}} = g_{\mathrm{Na}} o_{\mathrm{Na}} (v - E_{\mathrm{Na}}), \tag{16}$$

where the open probability is given by

$$o_{\mathrm{Na}} = m^3 j, \tag{17}$$

and m and j are gating variables governed by equations of the form (15).

Late sodium current The formulation of the late sodium current, I_{NaL}, is based on [14] and is given by

$$I_{\mathrm{NaL}} = g_{\mathrm{NaL}} o_{\mathrm{NaL}} (v - E_{\mathrm{Na}}), \tag{18}$$

where the open probability is given by

$$o_{\mathrm{NaL}} = m_L h_L, \tag{19}$$

and m_L and h_L are gating variables governed by equations of the form (15).

Transient outward potassium current The formulation of the transient outward potassium current, I_{to}, is based on [3] and is given by

$$I_{\mathrm{to}} = g_{\mathrm{to}} o_{\mathrm{to}} (v - E_{\mathrm{to}}), \tag{20}$$

where the open probability is given by

$$o_{\mathrm{to}} = q_{\mathrm{to}} r_{\mathrm{to}}, \tag{21}$$

and q_{to} and r_{to} are gating variables governed by equations of the form (15).

Rapidly activating potassium current The formulation of the rapidly activating potassium current, I_{Kr}, is based on [3] and is given by

$$I_{\mathrm{Kr}} = g_{\mathrm{Kr}} \sqrt{\frac{[K^+]_e}{5.4\ \mathrm{mM}}} o_{\mathrm{Kr}} (v - E_{\mathrm{K}}), \tag{22}$$

where

$$o_{\mathrm{Kr}} = x_{\mathrm{Kr1}} x_{\mathrm{Kr2}}, \tag{23}$$

and the dynamics of x_{Kr1} and x_{Kr2} are governed by equations of the form (15).

Slowly activating potassium current The formulation of the slowly activating potassium current, I_{Ks}, is based on [13] and is given by

$$I_{\mathrm{Ks}} = g_{\mathrm{Ks}} o_{\mathrm{Ks}} (v - E_{\mathrm{Ks}}), \tag{24}$$

where

$$o_{\mathrm{Ks}} = x_{\mathrm{Ks}}^2, \tag{25}$$

and the dynamics of x_{Ks} is governed by an equation of the form (15).

Inward rectifier potassium current The formulation of the inward rectifier potassium current, I_{K1}, is based on [13] and is given by

$$I_{\mathrm{K1}} = g_{\mathrm{K1}} \sqrt{\frac{[K^+]_e}{5.4\ \mathrm{mM}}} o_{\mathrm{K1}} (v - E_{\mathrm{K}}), \tag{26}$$

where

$$o_{\mathrm{K1}} = \frac{a_{\mathrm{K1}}}{a_{\mathrm{K1}} + b_{\mathrm{K1}}}, \tag{27}$$

$$a_{\mathrm{K1}} = \frac{1}{1 + e^{0.2(v - E_K - 59)}}, \tag{28}$$

$$b_{\mathrm{K1}} = \frac{0.5 e^{0.08(v - E_K + 5)} + e^{0.06(v - E_K - 594)}}{1 + e^{-0.5(v - E_K + 5)}}. \tag{29}$$

Hyperpolarization activated funny current The formulation for the hyperpolarization activated funny current, I_{f}, is based on [3] and is given by

$$I_{\mathrm{f}} = g_{\mathrm{f}} o_{\mathrm{f}} (v - E_{\mathrm{f}}), \tag{30}$$

where

$$o_{\mathrm{f}} = x_{\mathrm{f}}, \tag{31}$$

and the dynamics of x_{f} is governed by an equation of the form (15).

L-type Ca^{2+} current The formualtion for the L-type Ca^{2+} current, I_{CaL}, is based on the formulation in [13] and is given by

$$I_{\mathrm{CaL}} = n g_{\mathrm{CaL}} o_{\mathrm{CaL}} \frac{(2F)^2 v}{RT} \frac{0.341 c_d e^{\frac{2Fv}{RT}} - 0.341 c_e}{e^{\frac{2Fv}{RT}} - 1}, \tag{32}$$

where

$$o_{\mathrm{CaL}} = d f (1 - f_{\mathrm{Ca}}), \tag{33}$$

and the dynamics of d, f and f_{Ca} are governed by equations of the form (15). Furthermore, n is the scaling factor for the number of L-type calcium channels in the cell membrane. In the paper, we consider two alternative models for this factor, one model directly based on [1, 2] given by (9)–(10) and one updated model described in the main paper and repeated in (69)–(71) below.

Background currents The formulation of the background currents, I_{bCa} and I_{bCl}, are based on [13] and are given by

$$I_{\mathrm{bCa}} = g_{\mathrm{bCa}}(v - E_{\mathrm{Ca}}), \tag{34}$$

$$I_{\mathrm{bCl}} = g_{\mathrm{bCl}}(v - E_{\mathrm{Cl}}). \tag{35}$$

Sodium-calcium exchanger The formulation of the Na^+-Ca^{2+} exchanger current, I_{NaCa}, is based on [13] and is given by

$$I_{\mathrm{NaCa}} = \bar{I}_{\mathrm{NaCa}} \frac{e^{\frac{\nu F v}{RT}}[\mathrm{Na}^+]_i^3 c_e - e^{\frac{(\nu-1)Fv}{RT}}[\mathrm{Na}^+]_e^3 c_{sl}}{s_{\mathrm{NaCa}}\left(1 + \left(\frac{K_{\mathrm{act}}}{c_{sl}}\right)^2\right)\left(1 + k_{\mathrm{sat}} e^{\frac{(\nu-1)Fv}{RT}}\right)}, \tag{36}$$

where

$$s_{\mathrm{NaCa}} = K_{\mathrm{Ca},i}[\mathrm{Na}^+]_e^3\left(1 + \left(\frac{[\mathrm{Na}^+]_i}{K_{\mathrm{Na},i}}\right)^3\right) + K_{\mathrm{Na},e}^3 c_{sl}\left(1 + \frac{c_{sl}}{K_{\mathrm{Ca},i}}\right)$$
$$+ K_{\mathrm{Ca},e}[\mathrm{Na}^+]_i^3 + [\mathrm{Na}^+]_i^3 c_e + [\mathrm{Na}^+]_e^3 c_{sl}.$$

Sarcolemmal Ca^{2+} pump The formulation of the current through the sarcolemmal Ca^{2+} pump, I_{pCa}, is based on [13] and is given by

$$I_{\mathrm{pCa}} = \bar{I}_{\mathrm{pCa}} \frac{c_{sl}^2}{K_{\mathrm{pCa}}^2 + c_{sl}^2}. \tag{37}$$

Sodium-potassium pump The current through the Na^+-K^+ pump, I_{NaK}, is based on [13] and is given by

$$I_{\mathrm{NaK}} = \bar{I}_{\mathrm{NaK}} \frac{f_{\mathrm{NaK}}}{1 + \left(\frac{K_{\mathrm{Na,i}}^{\mathrm{NaK}}}{[\mathrm{Na}^+]_{\mathrm{i}}}\right)^4} \frac{[\mathrm{K}^+]_{\mathrm{e}}}{[\mathrm{K}^+]_{\mathrm{e}} + K_{\mathrm{K,e}}}, \tag{38}$$

where

$$f_{\mathrm{NaK}} = \frac{1}{1 + 0.12 e^{-0.1\frac{Fv}{RT}}} + \frac{0.037}{7}\left(e^{\frac{[\mathrm{Na}^+]_{\mathrm{e}}}{67}} - 1\right) e^{-\frac{Fv}{RT}}. \tag{39}$$

Nernst equilibrium potentials

The Nernst equilibrium potentials for the ion channels are defined as

$$E_{\mathrm{Na}} = \frac{RT}{F}\log\left(\frac{[\mathrm{Na}^+]_\mathrm{e}}{[\mathrm{Na}^+]_\mathrm{i}}\right), \tag{40}$$

$$E_{\mathrm{Ca}} = \frac{RT}{2F}\log\left(\frac{[\mathrm{Ca}^{2+}]_\mathrm{e}}{c_{sl}}\right), \tag{41}$$

$$E_{\mathrm{K}} = \frac{RT}{F}\log\left(\frac{[\mathrm{K}^+]_\mathrm{e}}{[\mathrm{K}^+]_\mathrm{i}}\right), \tag{42}$$

$$E_{\mathrm{Ks}} = \frac{RT}{F}\log\left(\frac{[\mathrm{K}^+]_\mathrm{e} + 0.018[\mathrm{Na}^+]_\mathrm{e}}{[\mathrm{K}^+]_\mathrm{i} + 0.018[\mathrm{Na}^+]_\mathrm{i}}\right), \tag{43}$$

$$E_{\mathrm{Cl}} = \frac{RT}{F}\log\left(\frac{[\mathrm{Cl}^+]_\mathrm{e}}{[\mathrm{Cl}^+]_\mathrm{i}}\right), \tag{44}$$

$$E_{\mathrm{f}} = -17 \text{ mV}, \tag{45}$$

for the parameter values given in Supplementary Table 3.

Ca^{2+} dynamics

The Ca^{2+} dynamics are governed by

$$\frac{dc_d}{dt} = \frac{1}{V_d}(J_{\mathrm{CaL}} - J_d^b - J_d^c), \qquad \frac{db_d}{dt} = \frac{1}{V_d}J_d^b, \tag{46}$$

$$\frac{dc_{sl}}{dt} = \frac{1}{V_{sl}}(J_e^{sl} - J_{sl}^c - J_{sl}^b + J_s^{sl}), \qquad \frac{db_{sl}}{dt} = \frac{1}{V_{sl}}J_{sl}^b, \tag{47}$$

$$\frac{dc_c}{dt} = \frac{1}{V_c}(J_{sl}^c + J_d^c - J_c^n - J_c^b), \qquad \frac{db_c}{dt} = \frac{1}{V_c}J_c^b, \tag{48}$$

$$\frac{dc_s}{dt} = \frac{1}{V_s}(J_n^s - J_s^{sl} - J_s^b), \qquad \frac{db_s}{dt} = \frac{1}{V_s}J_s^b, \tag{49}$$

$$\frac{dc_n}{dt} = \frac{1}{V_n}(J_c^n - J_n^s), \qquad \frac{db_t}{dt} = \frac{1}{V_c}J_t^b, \tag{50}$$

where c_d is the concentration of free Ca^{2+} in the dyad, b_d is the concentration of Ca^{2+} bound to a buffer in the dyad, c_{sl} is the concentration of free Ca^{2+} in the SL compartment, b_{sl} is the concentration of Ca^{2+} bound to a buffer in the SL compartment, c_c is the concentration of free Ca^{2+} in the bulk cytosol, b_c is the concentration of Ca^{2+} bound to a buffer that is not troponin in the bulk cytosol, b_t is the concentration of Ca^{2+} bound to troponin in the bulk cytosol, c_s is the concentration of free Ca^{2+} in the jSR, b_s is the concentration

of Ca^{2+} bound to a buffer in the jSR, and c_n is the concentration of free Ca^{2+} in the nSR. The expressions for the fluxes are specified below.

Ca^{2+} fluxes

Flux through the SERCA pumps The flux from the bulk cytosol to the nSR through the SERCA pumps is given by

$$J_c^n = \bar{J}_{\mathrm{SERCA}} \frac{\left(\frac{c_c}{K_c}\right)^2 - \left(\frac{c_n}{K_n}\right)^2}{1 + \left(\frac{c_c}{K_c}\right)^2 + \left(\frac{c_n}{K_n}\right)^2}. \tag{51}$$

Flux through the RyRs The flux from the jSR to the SL compartment is given by

$$J_s^{sl} = J_{\mathrm{RyR}} + J_{\mathrm{leak}}, \tag{52}$$

where J_{RyR} represents the flux through the active RyR channels and J_{leak} represents the flux through the RyR channels that are always open, given by

$$J_{\mathrm{RyR}} = p \cdot r \cdot \alpha_{\mathrm{RyR}}(c_s - c_{sl}), \tag{53}$$

$$J_{\mathrm{leak}} = \gamma_{\mathrm{RyR}} \cdot \alpha_{\mathrm{RyR}}(c_s - c_{sl}), \tag{54}$$

respectively. Here, p is the open probability of the active RyR channels given by

$$p = \frac{c_d^3}{c_d^3 + \kappa_{\mathrm{RyR}}^3}, \tag{55}$$

and r represents the fraction of RyR channels that are not inactivated and is governed by the equation

$$\frac{dr}{dt} = -\frac{J_{\mathrm{RyR}}}{\beta_{\mathrm{RyR}}} + \frac{\eta_{\mathrm{RyR}}}{p}(1 - r). \tag{56}$$

Passive diffusion fluxes between compartments The passive diffusion fluxes between compartments are given by

$$J_d^c = \alpha_d^c(c_d - c_c), \tag{57}$$

$$J_{sl}^c = \alpha_{sl}^c(c_{sl} - c_c), \tag{58}$$

$$J_n^s = \alpha_n^s(c_n - c_s). \tag{59}$$

Buffer fluxes The fluxes of free Ca^{2+} binding to a Ca^{2+} buffer are given by

$$J_d^b = V_d(k_{\text{on}}^d c_d(B_{\text{tot}}^d - b_d) - k_{\text{off}}^d b_d), \tag{60}$$
$$J_{sl}^b = V_{sl}(k_{\text{on}}^{sl} c_{sl}(B_{\text{tot}}^{sl} - b_{sl}) - k_{\text{off}}^{sl} b_{sl}), \tag{61}$$
$$J_c^b = V_c(k_{\text{on}}^c c_c(B_{\text{tot}}^c - b_c) - k_{\text{off}}^c b_c), \tag{62}$$
$$J_s^b = V_s(k_{\text{on}}^s c_s(B_{\text{tot}}^s - b_s) - k_{\text{off}}^s b_s), \tag{63}$$
$$J_t^b = V_c(k_{\text{on}}^t c_c(B_{\text{tot}}^t - b_t) - k_{\text{off}}^t b_t). \tag{64}$$

Membrane fluxes The membrane fluxes, J_{CaL}, J_{bCa}, J_{pCa}, and J_{NaCa}, are given by

$$J_{\text{CaL}} = -\frac{\chi C_m}{2F} I_{\text{CaL}}, \qquad J_{\text{pCa}} = -\frac{\chi C_m}{2F} I_{\text{pCa}}, \tag{65}$$
$$J_{\text{bCa}} = -\frac{\chi C_m}{2F} I_{\text{bCa}}, \qquad J_{\text{NaCa}} = \frac{\chi C_m}{F} I_{\text{NaCa}}, \tag{66}$$

where I_{CaL}, I_{bCa}, I_{pCa}, and I_{NaCa} are defined by the expressions given above. Furthermore,

$$J_e^{sl} = J_{\text{NaCa}} + J_{\text{pCa}} + J_{\text{bCa}}. \tag{67}$$

Na^+ dynamics

For the intracellular Na^+ concentration, we use the same approach as in [12]. In this approach, spatial gradients of $[Na^+]_i$ in the cell are ignored and the concentration is governed by

$$\frac{d[\text{Na}]_i}{dt} = -\frac{\chi C_m}{F} \left(I_{\text{Na}} + I_{\text{NaL}} + 3I_{\text{NaK}} + 3I_{\text{NaCa}} + 0.3293 I_{\text{f}} \right), \tag{68}$$

where the currents I_{Na}, I_{NaL}, I_{NaK}, I_{NaCa}, and I_{f} are specified above.

Protein regulation

The scaling factor n for the number of L-type calcium channels on the cell membrane is modeled by

$$\tau_n \frac{dn}{dt} = (c^* - c) H(c, n), \tag{69}$$
$$H(c, n) = h(n, n_-, \varepsilon_n) h(c, c^*, \varepsilon_c) + h(n_+, n, \varepsilon_n) h(c^*, c, \varepsilon_c), \tag{70}$$
$$h(a, b, \varepsilon) = \frac{1}{2} \left(1 + \tanh \left(\frac{a - b}{\varepsilon} \right) \right). \tag{71}$$

Parameter	Description	Value
V_d	Volume fraction of the dyadic subspace	0.001
V_{sl}	Volume fraction of the SL compartment	0.028
V_c	Volume fraction of the bulk cytosol	0.917
V_s	Volume fraction of the jSR	0.004
V_n	Volume fraction of the nSR	0.05
χ	Cell surface to volume ratio	0.9 μm^{-1}

Supplementary Table 2: Default geometry parameters of the base model.

Parameter	Description	Value
C_m	Specific membrane capacitance	0.01 pF/μm^2
F	Faraday's constant	96.485 C/mmol
R	Universal gas constant	8.314 J/(mol·K)
T	Temperature	310 K
$[Ca^{2+}]_e$	Extracellular Ca^{2+} concentration	1.8 mM
$[Na^+]_e$	Extracellular sodium concentration	155.3 mM
$[K^+]_e$	Extracellular potassium concentration	5.3 mM
$[K^+]_i$	Intracellular potassium concentration	59.5 mM
$[Cl^-]_e$	Extracellular chloride concentration	119.3 mM
$[Cl^-]_i$	Intracellular chloride concentration	15 mM

Supplementary Table 3: Physical constants and ionic concentrations of the base model.

Parameter	Value	Parameter	Value
g_{Na}	12.6 mS/μF	g_{CaL}	0.254 nL/(μF ms)
g_{NaL}	0.003 mS/μF	g_{bCa}	0.00007 mS/μF
g_{to}	0.1 mS/μF	$\bar{I}_{\mathrm{NaCa}}$	29.1 μA/μF
g_{Kr}	0.07 mS/μF	$\bar{I}_{\mathrm{pCa}}$	0.24 μA/μF
g_{Ks}	0.013 mS/μF	$\bar{J}_{\mathrm{SERCA}}$	0.00029 mM/ms
g_{K1}	0.088 mS/μF	α_{RyR}	0.0013 ms^{-1}
g_{f}	0.079 mS/μF	α_d^c	0.0038 ms^{-1}
g_{bCl}	0.012 mS/μF	α_{sl}^c	0.018 ms^{-1}
$\bar{I}_{\mathrm{NaK}}$	1.92 μA/μF	α_n^s	0.0093 ms^{-1}

Supplementary Table 4: Conductance values and similar parameters for each of the membrane currents and intracellular Ca^{2+} fluxes of the base model.

Parameter	Flux	Value
K_c	J_c^n	0.00025 mM
K_n	J_c^n	1.7 mM
β_{RyR}	J_s^{sl}	0.027 mM
γ_{RyR}	J_s^{sl}	0.001
κ_{RyR}	J_{RyR}	0.015 mM
η_{RyR}	J_s^{sl}	0.00001 ms^{-1}

Supplementary Table 5: Parameters for the intracellular Ca^{2+} fluxes of the base model.

Parameter	Current	Value
k_{sat}	I_{NaCa}	0.3
ν	I_{NaCa}	0.3
K_{act}	I_{NaCa}	0.00015 mM
$K_{\text{Ca},i}$	I_{NaCa}	0.0036 mM
$K_{\text{Ca},e}$	I_{NaCa}	1.3 mM
$K_{\text{Na},i}$	I_{NaCa}	12.3 mM
$K_{\text{Na},e}$	I_{NaCa}	87.5 mM
$K_{\text{Na},i}^{\text{NaK}}$	I_{NaK}	11 mM
$K_{\text{K},e}$	I_{NaK}	1.5 mM
K_{pCa}	I_{pCa}	0.0005 mM

Supplementary Table 6: Parameters for the membrane currents of the base model.

Parameter	Compartment	Value
B_{tot}^{c}	Bulk cytosol	0.034 mM
k_{on}^{c}	Bulk cytosol	40 ms^{-1}mM^{-1}
k_{off}^{c}	Bulk cytosol	0.03 ms^{-1}
B_{tot}^{d}	Dyad	4.07 mM
k_{on}^{d}	Dyad	100 ms^{-1}mM^{-1}
k_{off}^{d}	Dyad	1 ms^{-1}
B_{tot}^{sl}	Subsarcolemmal space	0.66 mM
k_{on}^{sl}	Subsarcolemmal space	100 ms^{-1}mM^{-1}
k_{off}^{sl}	Subsarcolemmal space	0.15 ms^{-1}
B_{tot}^{s}	Junctional SR	27 mM
k_{on}^{s}	Junctional SR	100 ms^{-1}mM^{-1}
k_{off}^{s}	Junctional SR	39.6 ms^{-1}

Supplementary Table 7: Parameters for the Ca^{2+} buffers of the base model.

Parameter	Value	Parameter	Value
τ_n	400 mMms	c^*	$5.39 \cdot 10^{-5}$ mM
n_-	0.1	n_+	3
ε_n	0.01	ε_c	10^{-7} mM

Supplementary Table 8: Parameters for the regulation of the number of L-type calcium channels in the cell membrane.

Current	Gate	z_∞	α_z	β_z	τ_z
I_{Na}	m	$\frac{1}{(1+e^{(-57-v)/9})^2}$	$0.13e^{-((v+46)/16)^2}$	$0.06e^{-((v-5)/51)^2}$	$\alpha_m + \beta_m$
	j	$\frac{1}{(1+e^{(v+72)/7})^2}$	0, if $v \geq -40$<br>$\frac{\left(\begin{array}{c}-2.5 \cdot 10^4 e^{0.2v} \\ -7 \cdot 10^{-6} e^{-0.04v}\end{array}\right)(v+38)}{1+e^{0.3(v+79)}}$, otherwise	$\frac{0.6e^{0.06v}}{1+e^{-0.1(v+32)}}$, if $v \geq -40$<br>$\frac{0.02e^{-0.01v}}{1+e^{-0.14(v+40)}}$, otherwise	$\frac{1}{\alpha_j + \beta_j}$
I_{NaL}	m_L	$\frac{1}{1+e^{(-43-v)/5}}$	$\frac{1}{6.8e^{(v+12)/35}}$	$8.6e^{-(v+77)/6}$	$\alpha_m + \beta_m$
	h_L	$\frac{1}{1+e^{(v+88)/7.5}}$			200 ms
I_{CaL}	d	$\frac{1}{1+e^{-(v+5)/6}}$	$\frac{1-e^{-\frac{v+5}{6}}}{0.035(v+5)}$		$\alpha_d \cdot d_\infty$
	f	$\frac{1}{1+e^{(v+35)/9}} + \frac{0.6}{1+e^{(50-v)/20}}$	$\frac{1}{0.02e^{-(0.034(v+14.5)^2)}+0.02}$		α_f
	f_{Ca}	$\frac{1.7c_d}{1.7c_d+0.012}$	$\frac{1}{1.7c_d+0.012}$		α_{Ca}
I_{to}	q_{to}	$\frac{1}{1+e^{(v+53)/13}}$	$\frac{39}{0.57e^{-0.08(v+44)}+0.065e^{0.1(v+46)}}$	6	$\alpha_{q_{\mathrm{to}}} + \beta_{q_{\mathrm{to}}}$
	r_{to}	$\frac{1}{1+e^{-(v-22.3)/18.75}}$	$\frac{14.4}{e^{0.09(v+30.61)}+0.37e^{-0.12(v+24)}}$	2.75	$\alpha_{r_{\mathrm{to}}} + \beta_{r_{\mathrm{to}}}$
I_{Kr}	x_{Kr1}	$\frac{1}{1+e^{-(v+20.7)/4.9}}$	$\frac{450}{1+e^{-(v+45)/10}}$	$\frac{6}{1+e^{(v+30)/11.5}}$	$\alpha_{x_{\mathrm{Kr1}}} \cdot \beta_{x_{\mathrm{Kr1}}}$
	x_{Kr2}	$\frac{1}{1+e^{(v+88)/50}}$	$\frac{3}{1+e^{-(v+60)/20}}$	$\frac{1.12}{1+e^{(v-60)/20}}$	$\alpha_{x_{\mathrm{Kr2}}} \cdot \beta_{x_{\mathrm{Kr2}}}$
I_{Ks}	x_{Ks}	$\frac{1}{1+e^{-(v+3.8)/14}}$	$\frac{990}{1+e^{-(v+2.4)/14}}$		$\alpha_{x_{\mathrm{Ks}}}$
I_{f}	x_{f}	$\frac{1}{1+e^{(v+78)/5}}$	$\frac{1900}{1+e^{(v+15)/10}}$		$\alpha_{x_{\mathrm{Ks}}}$

Supplementary Table 9: Specification of the parameters z_∞ and τ_z, for $z = m$, j, m_L, h_L, d, f, f_{Ca}, q_{to}, r_{to}, x_{Kr1}, x_{Kr2}, x_{Ks} and x_{f} in the equations for the gating variables (15).

Supplementary References

[1] Timothy O'Leary, Alex H Williams, Alessio Franci, and Eve Marder. Cell types, network homeostasis, and pathological compensation from a biologically plausible ion channel expression model. *Neuron*, 82(4):809–821, 2014.

[2] Nicolae Moise and Seth H Weinberg. Emergent activity, heterogeneity, and robustness in a calcium feedback model of the sinoatrial node. *Biophysical Journal*, 122(9):1613–1632, 2023.

[3] Michelangelo Paci, Jari Hyttinen, Katriina Aalto-Setälä, and Stefano Severi. Computational models of ventricular-and atrial-like human induced pluripotent stem cell derived cardiomyocytes. *Annals of Biomedical Engineering*, 41(11):2334–2348, 2013.

[4] Divya C Kernik, Stefano Morotti, HaoDi Wu, Priyanka Garg, Henry J Duff, Junko Kurokawa, José Jalife, Joseph C Wu, Eleonora Grandi, and Colleen E Clancy. A computational model of induced pluripotent stem-cell derived cardiomyocytes incorporating experimental variability from multiple data sources. *The Journal of Physiology*, 597(17):4533–4564, 2019.

[5] Stefano Severi, Matteo Fantini, Lara A Charawi, and Dario DiFrancesco. An updated computational model of rabbit sinoatrial action potential to investigate the mechanisms of heart rate modulation. *The Journal of Physiology*, 590(18):4483–4499, 2012.

[6] James Kramer, Carlos A Obejero-Paz, Glenn Myatt, Yuri A Kuryshev, Andrew Bruening-Wright, Joseph S Verducci, and Arthur M Brown. MICE models: superior to the HERG model in predicting Torsade de Pointes. *Scientific Reports*, 3(1):1–7, 2013.

[7] Lucia Romero, Jordi Cano, Julio Gomis-Tena, Beatriz Trenor, Ferran Sanz, Manuel Pastor, and Javier Saiz. In silico QT and APD prolongation assay for early screening of drug-induced proarrhythmic risk. *Journal of Chemical Information and Modeling*, 58(4):867–878, 2018.

[8] Antonella Di Stilo, Sonja Visentin, Clara Cena, Andrea Marcello Gasco, Giuseppe Ermondi, and Alberto Gasco. New 1, 4-dihydropyridines conjugated to furoxanyl moieties, endowed with both nitric oxide-like and calcium channel antagonist vasodilator activities. *Journal of Medicinal Chemistry*, 41(27):5393–5401, 1998.

[9] John K Gibson, Yimei Yue, Jared Bronson, Cassie Palmer, and Randy Numann. Human stem cell-derived cardiomyocytes detect drug-mediated changes in action potentials and ion currents. *Journal of Pharmacological and Toxicological Methods*, 70(3):255–267, 2014.

[10] Junyi Ma, Liang Guo, Steve J. Fiene, Blake D. Anson, James A. Thomson, Timothy J. Kamp, Kyle L. Kolaja, Bradley J. Swanson, and Craig T. January. High purity human-induced pluripotent stem cell-derived cardiomyocytes: electrophysiological properties of action potentials and ionic currents. *American Journal of Physiology-Heart and Circulatory Physiology*, 301(5):H2006–H2017, 2011. PMID: 21890694.

[11] Karoline H Jæger, Verena Charwat, Bérénice Charrez, Henrik Finsberg, Mary M Maleckar, Sam Wall, Kevin Healy, and Aslak Tveito. Improved computational identification of drug response using optical measurements of human stem cell derived cardiomyocytes in microphysiological systems. *Frontiers in Pharmacology*, 10:1648, 2020.

[12] Karoline H Jæger, Samuel Wall, and Aslak Tveito. Computational prediction of drug response in short QT syndrome type 1 based on measurements of compound effect in stem cell-derived cardiomyocytes. *PLoS Computational Biology*, 17(2):e1008089, 2021.

[13] Eleonora Grandi, Francesco S Pasqualini, and Donald M Bers. A novel computational model of the human ventricular action potential and Ca transient. *Journal of Molecular and Cellular Cardiology*, 48(1):112–121, 2010.

[14] Thomas O'Hara, László Virág, András Varró, and Yoram Rudy. Simulation of the undiseased human cardiac ventricular action potential: Model formulation and experimental validation. *PLoS Computational Biology*, 7(5):e1002061, 2011.
